# High-performance waveguide coupled Germanium-on-silicon single-photon avalanche diode with independently controllable absorption and multiplication

**DOI:** 10.1515/nanoph-2022-0663

**Published:** 2023-02-14

**Authors:** Heqing Wang, Yang Shi, Yan Zuo, Yu Yu, Lei Lei, Xinliang Zhang, Zhengfang Qian

**Affiliations:** Key Laboratory of Optoelectronic Devices and Systems of Ministry of Education and Guangdong Province, College of Physics and Optoelectronic Engineering, Shenzhen University, Shenzhen 518060, China; Wuhan National Laboratory for Optoelectronics, School of Optical and Electrical Information, Huazhong University of Science and Technology, Wuhan 430074, China

**Keywords:** detectors, integrated optics devices, single photon avalanche diodes

## Abstract

Germanium-on-silicon (Ge-on-Si) single photon avalanche diodes (SPADs) have received wide attention in recent years due to their potential to be integrated with Si photonics. In this work, we propose and demonstrate a high-performance waveguide coupled Ge-on-Si separate-absorption-charge-multiplication SPAD with three electric terminals. By providing two separate voltage drops on the light absorption and multiplication regions, the drift and multiplication of carriers can be optimized separately. This indeed improves the freedom of voltage regulation for both areas. Moreover, thanks to the separate controlling, doping profile of the charge layer is greatly released compared to that of the conventional device because of the flexible carrier injection. In this scenario, the dark counts of the detector can be largely reduced through decreasing the electric field on the sidewalls of the Ge absorption region without affecting the detection efficiency. The proposed SPAD exhibits a high on-chip single photon detection efficiency of 34.62% and low dark count rates of 279 kHz at 1310 nm with the temperature of 78 K. The noise equivalent power is as low as 3.27 × 10^−16^ WHz^−1/2^, which is, to the best of our knowledge, the lowest of that of the reported waveguide coupled Ge-on-Si SPADs. This three-terminal SPAD enables high-yield fabrication and provides robust performance in operation, showing a wide application prospect in applications such as on-chip quantum communication and lidar.

## 1 Introduction

Over the past decade, single photon detection technology has been brought to the fore as the rapid rise of weak optical signal recognition in optical communication and quantum information [1–3]. The photomultiplier tube (PMT) [4–7], single-photon avalanche diode (SPAD) [8–10], and superconducting nanowire single photon detector (SNSPD) [11–13] have been widely used because of their excellent single photon detection performance. PMTs are the first used for single-photon detection and can have large active areas, however, it is challenging to achieve a high detection efficiency while simultaneously remaining a low power consumption, high time-resolution, and low cost. SNSPDs are then proposed and experimentally demonstrated to perform improved detection efficiency. Even though SNSPD exhibits a lower dark count rates (DCR), higher photon count rates and more accurate time resolution compared to PMT, the cryogenic operating condition (typically below 10 K) is still regarded as the main obstacle to the practical development of SNSPD [14]. To overcome the intrinsic limitations of SNSPD, single photon avalanche diode (SPAD) has become the mainstream solution to the practical single photon detection since it provides a valid alternative under a more relaxed temperature condition from 78 K to room temperature. This improvement significantly optimizes the size, weight, power consumption, and cost of the device [15–18].

So far, there have been mature commercial products such as the Geiger mode InGaAs/InP SPADs, which have been widely used at the infrared wavelength band [10, 19, 20]. Due to the incompatibility with silicon-based integrated circuits, such detectors usually work as discrete devices or modules. On the other hand, Germanium (Ge) not only offers a sensitive response in the infrared, but is also well compatible with the CMOS technology for high-density and large-scale integrated photoelectronic products [21–24]. These intrinsic advantages lead to dramatic interests to the Ge-on-silicon (Ge-on-Si) SPADs. The reported Ge-on-Si SPADs can be divided into two categories with different optical coupling methods: normal-incidence and the waveguide-coupled. Comparatively, the latter is more beneficial to integrate with other photonic devices to perform the on-chip system. The first demonstration of waveguide coupled Ge-on-Si absorption-charge-multiplication (SACM) SPAD was reported by Martinez et al., and the detector shows on-chip single photon detection efficiency (SPDE) of 27.09%, DCR of 534 kHz at 1310 nm and 80 K [25]. For a better performance, the key research is to reduce the DCR of the detector, which is mainly contributed by the high density of interface traps at the exposed sidewalls [26–32]. Great efforts, such as surface passivation [33] and electric field localization [34], were proposed to suppress the density of interface traps and interface electric field on Ge. Ge-on-Si SACM structure with three electric terminals offers an alternative way to suppress the electric field of the interface traps, offering two separate voltage drops across the light absorption and charge multiplication regions [35–37]. The electric field of the interface on Ge can be reduced by independently controlling the voltage in the absorption region without affecting the avalanche probability in multiplication region. Although similar three-terminal structure had been used in high-speed avalanche photodiodes, it has not been utilized in high-performance SPADs.

In this paper, we present and experimentally demonstrate a Ge-on-Si SACM SPAD with three electric terminals. Compared with the conventional SACM structure which has only one bias voltage to simultaneously control the light absorption and charge multiplication regions, the proposed three-terminal SPAD provides two separate voltage drops for independent manipulations, enabling a more flexible and precise regulation of the voltage for both areas. Taking advantage of this design, the DCR of the detector can be greatly reduced by decreasing the electric field at the interface of Ge without affecting the SPDE. The proposed SPAD achieves a high on-chip SPDE of 34.62% while maintains a low DCR of 279 kHz at 1310 nm and 78 K, resulting in a NEP of 3.27 × 10^−16^ WHz^−1/2^. To the best of our knowledge, this is the lowest NEP among the reported waveguide coupled Ge-on-Si SPADs. These results illustrate clear potential for integration with Si photonics for on-chip applications.

## 2 Design and fabrication

**Figure 1:**
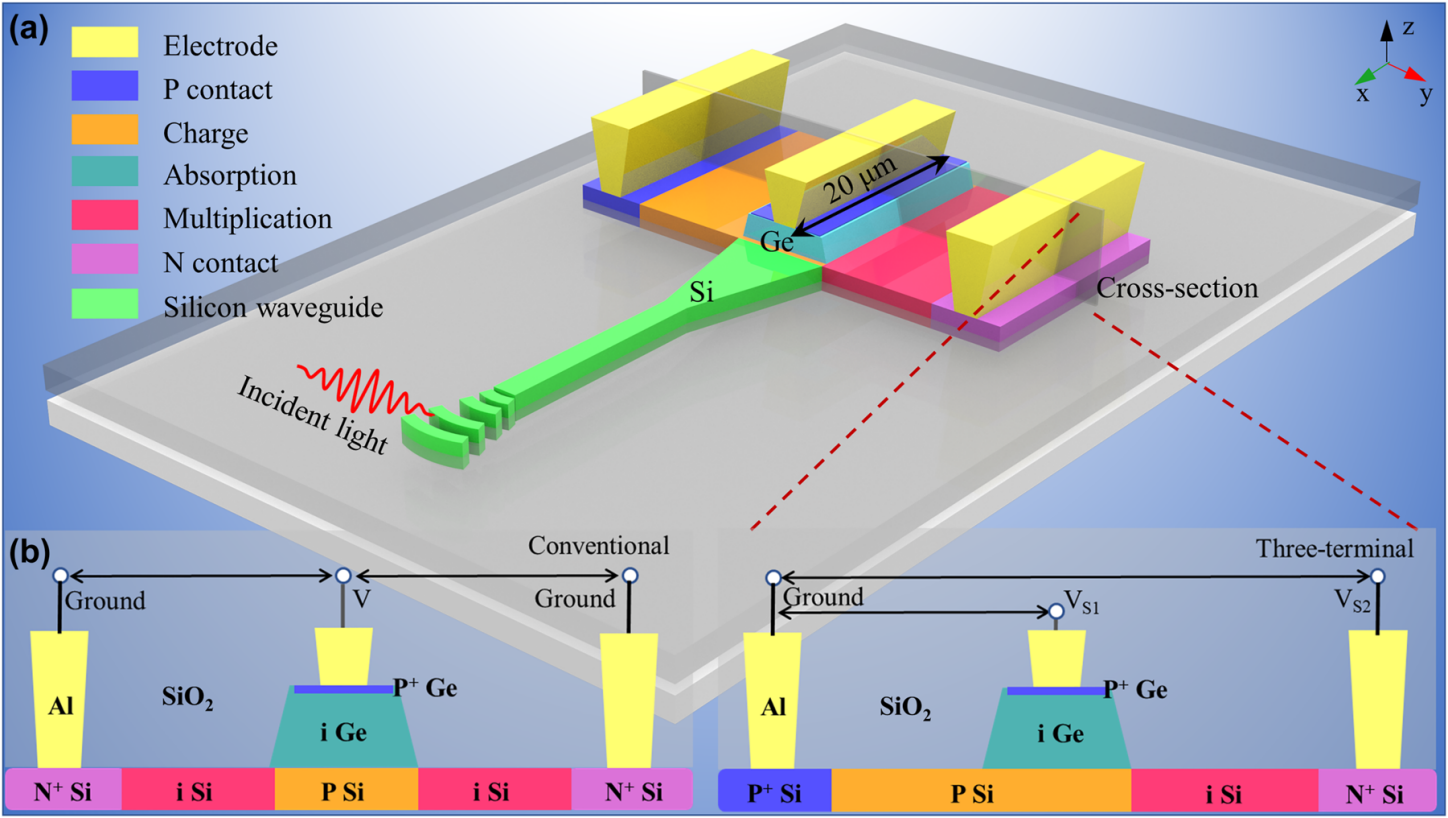
The structure of the wavegiude three-terminal SACM SPAD. (a) The 3D illustration of the waveguide three-terminal SACM SPAD. (b) The schematic cross-section diagrams of the conventional and three-terminal SACM SPAD. The figures are not to actual scale.

The 3D illustration of the proposed three-terminal SACM SPAD is shown in Figure 1(a). The incident light transmits from the channel Si waveguide to the Ge absorption region through a grating coupler. A 20-μm-length Ge guarantees a sufficient optical absorption at 1310 nm. The width and height of the Ge are 600 and 200 nm, respectively. The 800 nm wide intrinsic Si is designed for effective multiplication. A highly-doped shallow boron implantation is utilized to form an electric contact on the top of Ge, while the other two electric contacts are located on the P-doped and N-doped Si, resulting in the three-terminal structure. Typically, the average doping concentrations are controlled at the level of ∼1 × 10^20^ cm^−3^ for P contact, ∼1 × 10^17^ cm^−3^ for charge region, and ∼1 × 10^20^ cm^−3^ for N contact, respectively. In order to show the device structure more clearly, the schematic cross-section diagrams of conventional and three-terminal SACM SPAD are also shown in Figure 1(b) as a comparison.

The three electric terminals SPAD contains two junctions, one is between P-doped Ge and P-doped Si, and the other is between N-doped and P-doped Si. P-doped Si is set as ground, and voltages (i.e., *V*_S1_ and *V*_S2_) are loaded on the P-doped Ge and N-doped Si, respectively. Benefitting from this structure, two separate voltage drops can be controlled on the absorption and multiplication regions. More specifically, *V*_S1_ dynamically manipulates the electric field in the Ge layer, separates photon-generated electron-hole pairs and drives electrons toward charge multiplication, while *V*_S2_ is in charge of the electric field in the intrinsic silicon for multiplication. It is worth noting that being different from the conventional SACM structure which has only one bias voltage to simultaneously control the light absorption and charge multiplication regions, the proposed three-terminal SPAD provides two separate voltage drops for independent manipulations, offering a better regulation of the voltage for both areas. Moreover, thanks to the separate controlling, doping profile of the charge layer is greatly released compared to that of the conventional device because of the flexible carrier injection.

**Figure 2:**
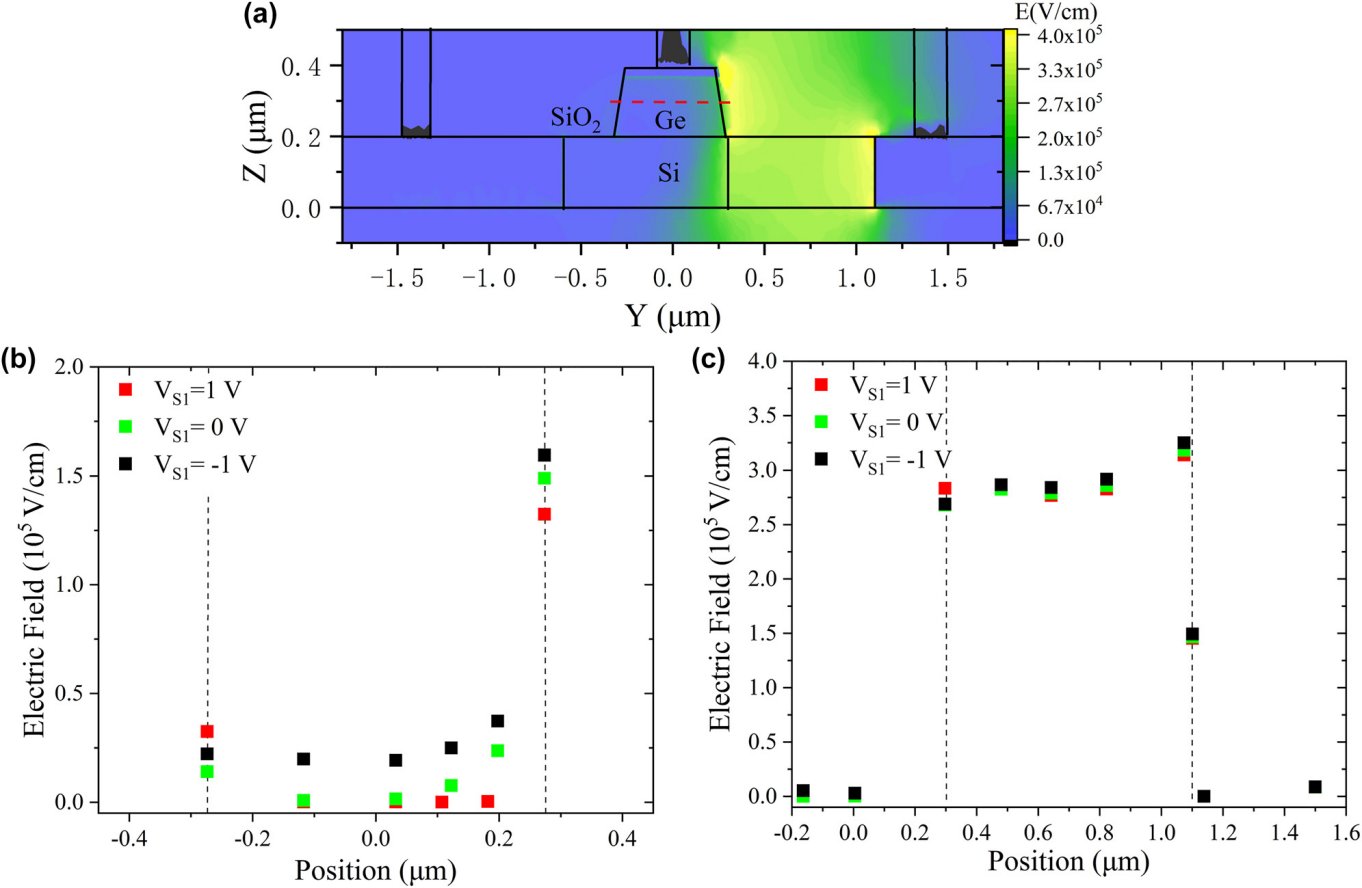
The electrical field distribution of the detector. (a) The simulated two-dimensional electric field distribution of the cross-section plane. (b) Simulated electrical field distribution along the longitudinal cross section at the center of the Ge layer with different *V*_S1_. (c) Simulated electrical field distribution of the multiplication layer with different *V*_S1_.

Figure 2(a) displays the simulated two-dimensional electric field distribution in the vertical cross-section plane. This electric field profile is shown for a potential held at near the avalanche breakdown voltage of *V*_S2_ without applying *V*_S1_. It can be seen that the electric field as high as 3.5 × 10^5^ V∕cm is generated in the intrinsic silicon regions, which is above the impact ionization field threshold for avalanche multiplication in silicon. And the electric field in the Ge absorption region keeps a lower level, suppressing undesired avalanche breakdown in Ge. Figure 2(b) shows the simulated electric field distribution at the center of the Ge layer along the transverse cross-section (i.e., the red dotted line shown in Figure 2(a)). The electric field in the Ge absorption region and the Ge-SiO_2_ interface decreases with the increase of *V*_S1_. Since the DCR is mainly associated with the high density of interface traps at the exposed sidewalls, increasing *V*_S1_ is beneficial to reduce the dark counts. Additionally, the electric field distribution of avalanche multiplication region is also analyzed in Figure 2(c). It is seen that with different *V*_S1_, the electric field almost overlaps; indicating *V*_S1_ nearly has no effect on the avalanche multiplication. In addition, by tuning *V*_S1_ to decrease the electric field on the sidewalls of the Ge absorption region, the DCR of the detector can be reduced without affecting the avalanche probability in multiplication region.

## 3 Device performance

**Figure 3:**
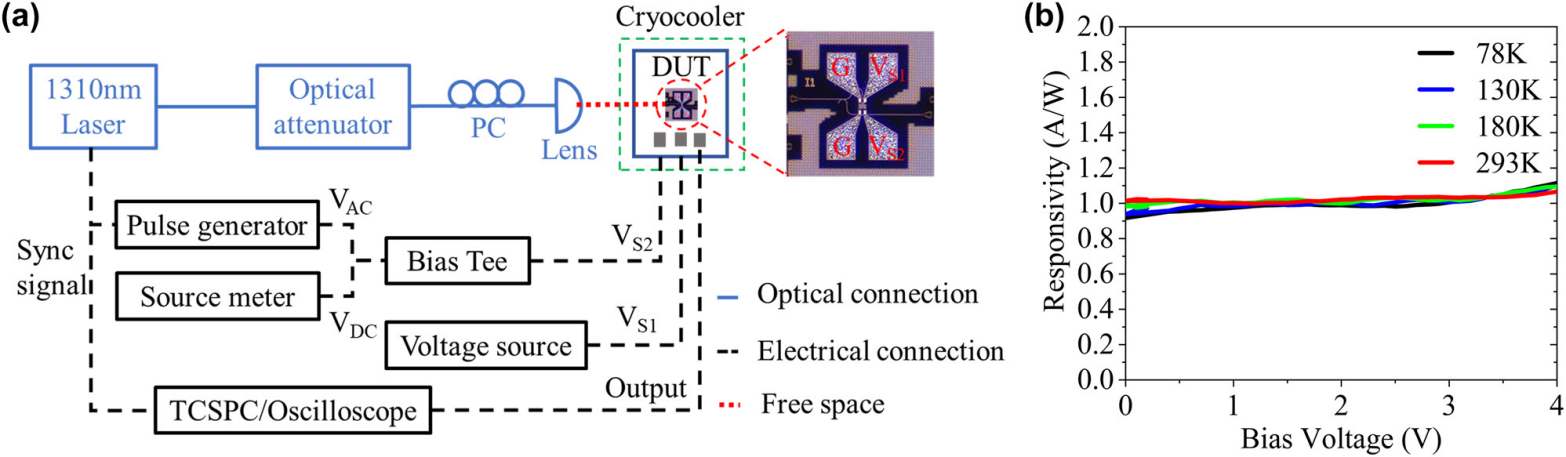
Experimental setup for the measurement. (a) Experimental setup for characterizing the performances of the three-terminal SACM SPAD. *V*_DC_ and *V*_AC_ are generated from a source meter and a pulse generator, respectively. *V*_S1_ is loaded by a separated power supply. The blue lines denote optical fiber connections, the black dotted lines denote cables for electrical connections and the red dotted line denotes the free-space optical connection. PC, polarization controller; DUT, device under test; TCSPC, time-correlated single photon counting. (b) The responsivity of the standard PIN detector at different temperatures.

The experimental setup is shown in Figure 3(a). Single photon detection of the SPAD is achieved by the gated Geiger-mode operation. This mode combines a DC voltage (*V*_DC_) just below breakdown and a series of periodic AC overbias (*V*_AC_) pulses through the bias-tee and loaded on *V*_S2_. We can evaluate the detection performance with the percent excess bias defined by the ratio *V*_ex_/*V*_br_, where *V*_br_ is the breakdown voltage of the device (defined as the voltage at the dark current reached 1 μA.) and *V*_ex_ is the voltage above *V*_br_. The device is wire-bonded to a PCB pad with SMP connectors and placed in a temperature-controlled liquid nitrogen cryocooler for adjusting the temperature from 293 K to 78 K. The output response is observed and recorded by a high-speed oscilloscope and a time-correlated single photon counting (TCSPC, PicoHarp 300). The optical pulsed waves synchronized with AC signals are generated from a laser with the wavelength of 1310 nm (EXPO OSICS T100). An optical attenuator (EXFO FVA-60B) followed by a polarization controller is utilized to optimize the incident optical power and polarization for optimal coupling efficiency. The optical signal is coupled into the SPAD via a grating coupler, assisting by a plano-convex lens. Particularly, the incident light is attenuated to the single-photon level with the average photon number <*n*> = 0.1. The optical power of incident light is calibrated by a standard Ge-Si PIN detector with the same designed grating coupler and fabricated on the same wafer. The coupling loss from lens to the grating coupler is measured as 13.2 dB, of which the insertion loss of the grating coupler is 4.3 dB. During the Geiger mode operation, the re-optimization of the coupling depends on the quality and number of triggers observed on the electric oscilloscope. Figure 3(b) shows the measured photon responsivity of the PIN detector at different temperatures, the internal responsivity (exclude coupling loss and waveguide propagation loss) is as high as ∼0.98 A/W. Based on the measured photocurrent of the detector, the light intensity entering the detector can be calculated, and then the incident photon power can be adjusted to the desired intensity by adjusting the optical attenuator. In this work, the on-chip SPDEs are measured by calibrating the optical power after coupled into the detector.

**Figure 4:**
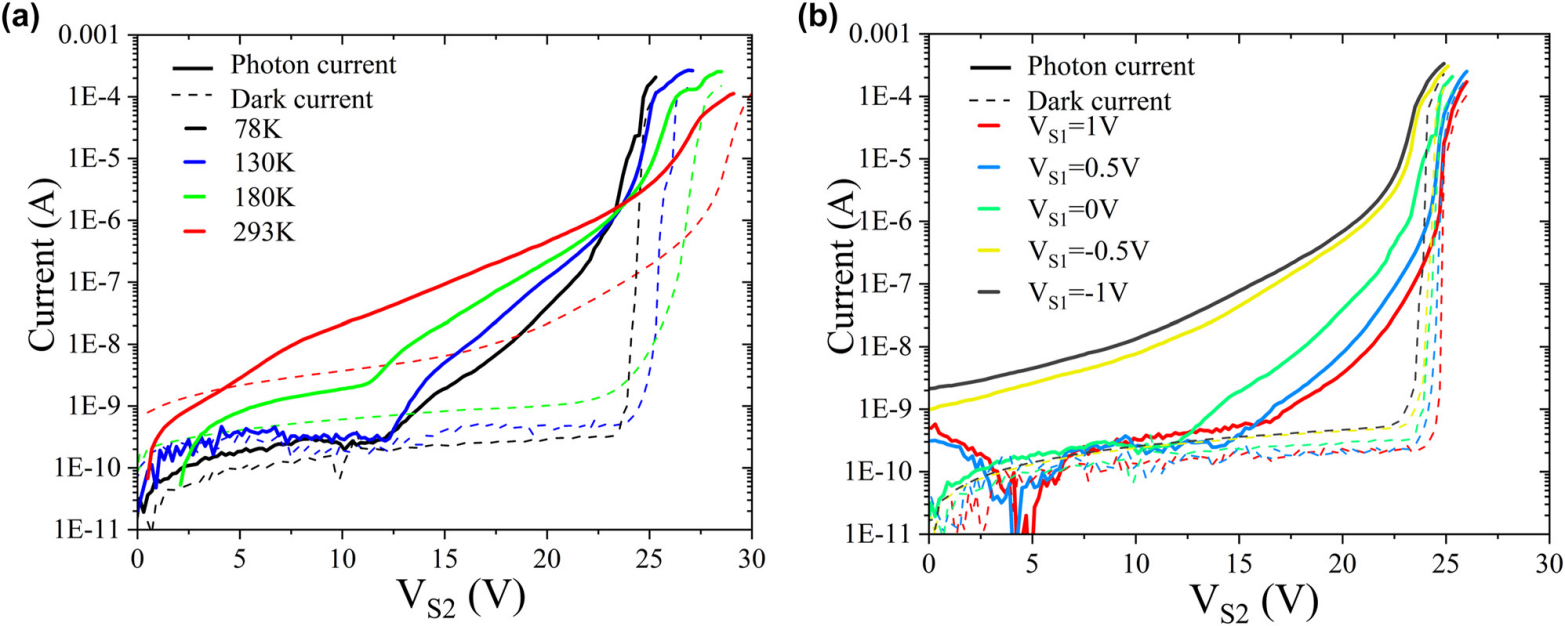
The measured *I*–*V* curves of the device. (a) The measured *I*–*V* curves of the device at temperature from 293 K to 78 K when the voltage *V*_S1_ = 0 V. The solid and dashed lines represent the photocurrent and dark current, respectively. (b) The *I*–*V* curves of the detector with different *V*_S1_ at 78 K.

Firstly, we measure the DC performances of the detector at temperature range from 293 K to 78 K. Figure 4(a) shows the dark and illuminated *I*–*V* characteristics of the device without applying *V*_S1_. The response exhibits a lower and progressively sharper breakdown voltage as the temperature decreases. This is benefit from the low temperature induced carrier cooling caused by the phonon scattering [38]. As the temperature is reduced, the characteristics demonstrate a lowering of dark current just prior to breakdown, showing a decrease in noise photon-counting behavior as the temperature decreases. We then measured the *I*–*V* response of the detector with different *V*_S1_ at 78 K, as shown in Figure 4(b). With the increasing reversed *V*_S1_, both photon and dark current rise due to the enhanced electric field of the Ge layer. On the contrary, for the increasing positive *V*_S1_, the electric field in Ge region is weakened, leading to the decrease of response performance. When *V*_S1_ is greater than 1 V, the electric field in Ge is almost ignorable, resulting in significantly lower responsivity. According to the above measurement, the optimal DCR of the proposed three-terminal SACM SPAD can be achieved with *V*_S1_ = 1 V at the temperature of 78 K since the DCR of the SPAD is proportional to the primary dark current [39].

Specifically, dark current in the detector are closely related to six major types of current, including: (1) the diffusion current (*I*_diff_), (2) the generation-recombination current governed by the SRH process (*I*_SRH_), (3) the generation-recombination current enhanced by trap-assisted-tunneling (*I*_TAT_), (4) the band-to-band tunneling current (*I*_BTBT_), (5) the avalanche current (*I*_AVA_) that occurs under high electric fields, and (6) the shunt resistance current (*I*_shunt_) [31]. Activation energy (*E*_a_) is then utilized to further describe the corresponding current [30, 40, 41]. Usually, *I*_dark_ ∞ exp(−*E*_a_/*kT*), where *k* is the Boltzmann constant and *T* is the temperature. The *E*_a_ of *I*_diff_ is identical to the bandgap (*E*_g_) of the material. In the case of Ge, *E*_a_ of the *I*_diff_ is about 0.66 eV. *I*_SRH_ has an *E*_a_ of half bandgap around 0.33 eV. *I*_TAT_ has smaller *E*_a_ less than 0.33 eV. *I*_BTBT_ and *I*_AVA_ are generated at high electric field >3 × 10^5^ V/cm, which is quite hard to reach in Ge in our simulation. *I*_shunt_ is given by *I*_shunt_ = *V*/*R*_s_, where *V* denotes the bias voltage *V*_S2_ and *R*_s_ is the shunt resistance. This current, which mainly includes the shunt current inside the device and leakage current in the test circuit, has a relatively small *E*_a_ and is insensitive to the temperature [42].

**Figure 5:**
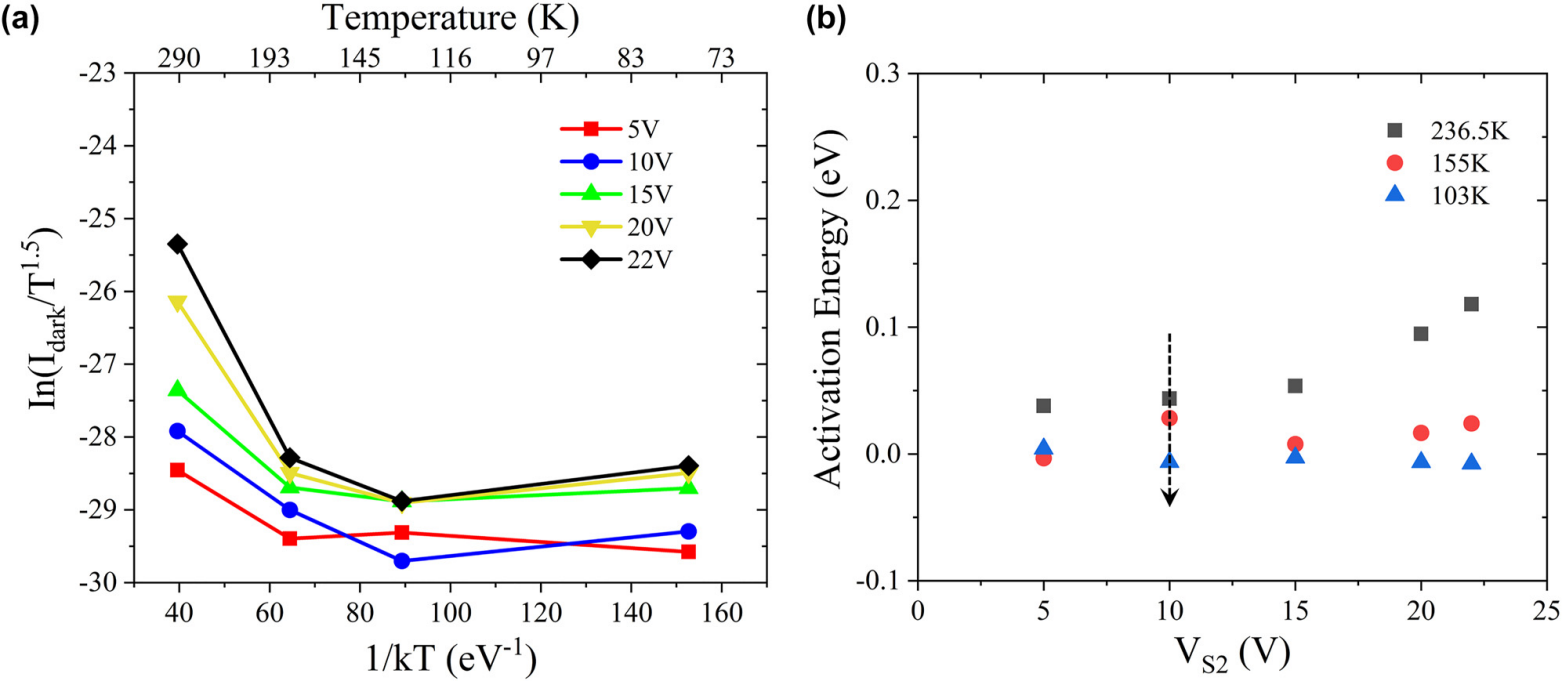
Dark current and activation energy calculation. (a) The Arrhenius plot of the *I*_dark_ as a function of 1/*kT* at various bias voltage *V*_S2_ under the temperature 293 K, 180 K, 130 K, and 78 K. (b) The evolution of *E*_a_ versus the bias voltage *V*_S2_.

Figure 5(a) displays the Arrhenius plot of the *I*_dark_ as a function of 1/*kT* at various bias voltage *V*_S2_ under the temperature of 293 K, 180 K, 130 K and 78 K. According to the expression mentioned above, *E*_a_ can be regarded as the slope of the curve, whose evolution is displayed as a function of bias voltage *V*_S2_ in Figure 5(b). One can see that all calculated *E*_a_ are lower than *E*_g_/2 of Ge (i.e., 0.33 eV), indicating that *I*_TAT_ and *I*_shunt_ dominates the dark current under different bias voltages. Meanwhile, at the same bias voltage of *V*_S2_, *E*_a_ declines with the decreasing temperature and nearly reaches zero which is associated with the gradually increasing proportion of *I*_shunt_ in the total dark current gradually increases. This also well explains the nearly linear variation of dark current observed in Figure 5(a) at low voltage at 77 K. Actually, the dark current at this temperature is close to the leakage current we measured in the circuit.

**Figure 6:**
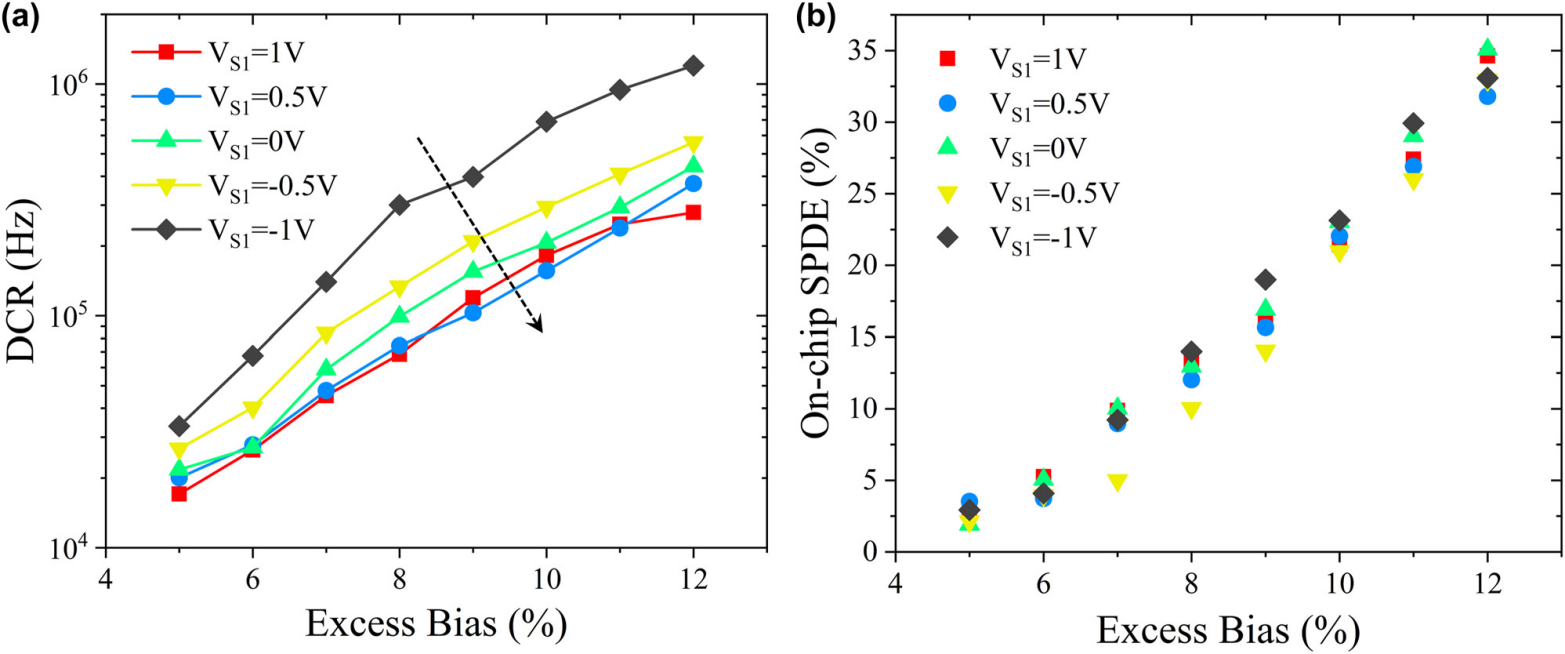
DCR and on-chip SPDE of the detector. (a) DCR of the detection system versus the excess bias with different *V*_S1_ at 78 K. (b) On-chip SPDE versus the excess bias with different *V*_S1_ at 78 K.

The DCR is then calculated by the equation [25]:

$$DCR=-\frac{1}{\tau }\ln\left(1-P_{\text{d}}\right),$$

where *P*_d_ denotes the dark count probability, which is measured by dividing the sum of dark count data over the repetition rate of gate signals. Here, the repetition rate of gated Geiger-mode is set to be 10 kHz with the pulse width *τ* of 10 ns. In Figure 6(a), we investigate the DCRs with the variable *V*_S1_ at 78 K. According to the measured response in Figure 4(b), the breakdown voltage is estimated to be 25 V at 78 K and *V*_S1_ = 1 V. The DC bias is set to 24 V, and the corresponding primary dark current is 0.21 nA. Similarly, the parameters with different *V*_S1_ can be selected. By applying 10 kHz repetition rate, no obvious afterpulse is captured on the oscilloscope, which means a low probability of afterpulse effects. Similar to the trend of dark current shown in Figure 4(b), the DCR of SPAD decreases with the increasing *V*_S1_ and reaches to the smallest when *V*_S1_ is biased at 1 V.

The SPDE is investigated according to [26]:

$$\eta =\frac{1}{\left\langle n\right\rangle }\ln\left(\frac{1-P_{\text{d}}}{1-P_{\text{c}}}\right),$$

where *P*_c_ is the total count probability with the light incidence, being calculated by dividing the sum of count data over the total number of gate signals. The SPDE depends mainly on the probability of photon absorption in Ge absorption region, electron drift from Ge absorption into Si multiplication region and successful avalanche trigger in Si multiplication region. Photon absorption remains almost constant at the same temperature. Electron drift to the Si multiplication region is influenced by the electric field in Ge, and the probability of successful avalanche trigger is related to the electric field in Si multiplication region. The electric field in multiplication region is proportional to the excess bias, in Figure 6(b) it can be observed that the SPDE increases nearly linearly with excess bias. The SPDE remains relatively constant from different *V*_S1_, indicating that modulating the electric field in Ge between −1 and 1 V does not affect the drift of electrons from the Ge absorption region towards the Si multiplication region, while we do observe a drop with efficiency when the applied voltage is greater than 1 V.

**Figure 7:**
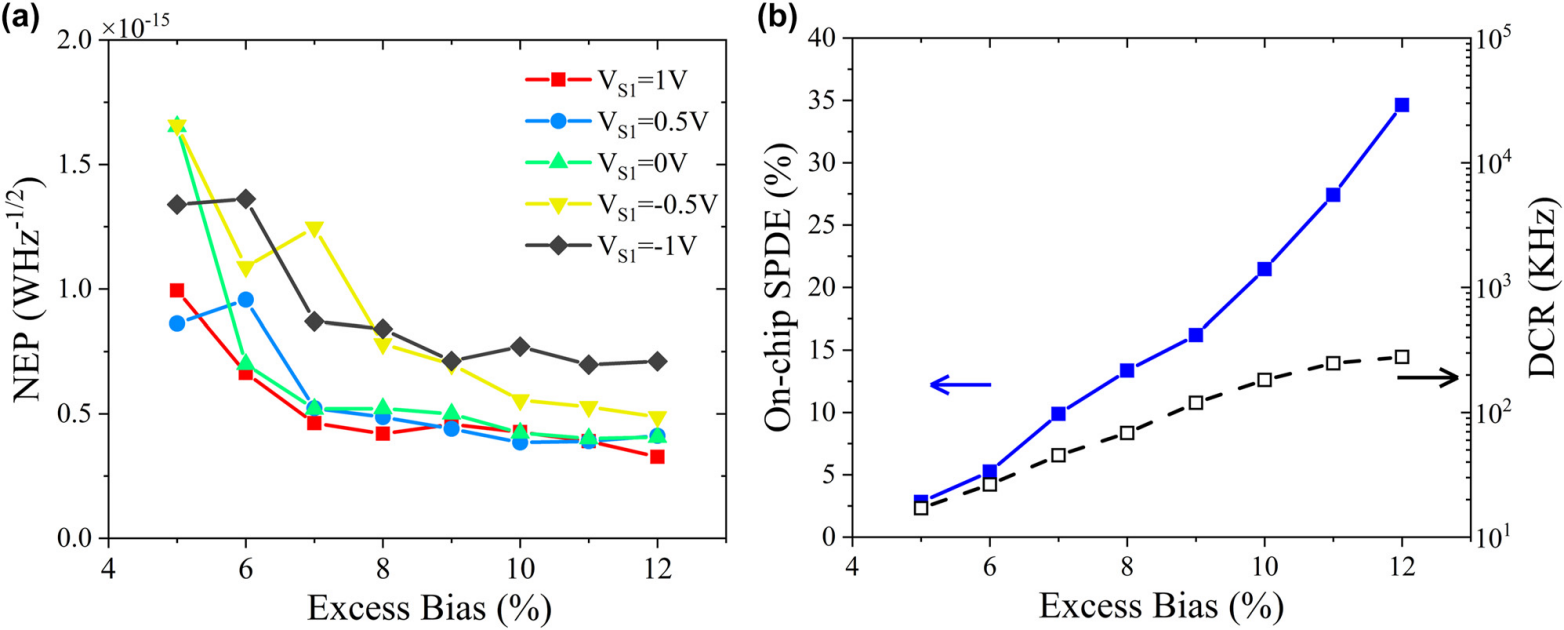
NEP and the best DCR and SPDE of the detector. (a) NEP versus excess bias with different voltage at 78 K. (b) The best feature of DCR and SPDE curve with applied voltage *V*_S1_ = 1 V.

NEP is another important index to evaluate the performance of a detector. Typically, it is defined as:

$$NEP=\frac{h\nu }{SPDE}\sqrt{2DCR},$$

where *h* is Planck’s constant, and *ν* is the photon frequency. As shown in Figure 7(a), the NEP is demonstrated as a function of excess bias with the variable *V*_S1_. Since the lowest DCR is achieved at *V*_S1_ = 1 V as shown in Figure 4(a), the detector presents the most attractive NEP with the same condition. Figure 7(b) concludes the best DCR and SPDE feature: the detector has a minimum NEP of 3.27 × 10^−16^ WHz^−1/2^ with excess bias of 12%, detection efficiency of 34.62%, and DCR of 279 kHz. More comparison between our device and the state-of-the-art achievements on Ge-on-Si and InGaAs/InP SPADs can be found in Table 1. Among the integrated waveguide coupled Ge-on-Si SPAD solutions, the proposed device provides the lowest DCR and NEP, illustrating clear potential for on-chip applications. Progress has been made with regards to the device performance on Ge-on-Si platform, but there is still some way to go compared with the commercial III–V SPADs. However, considering the integration compatibility with CMOS platform for manufacturing, Ge-on-Si solutions are still of great appealing.

**Table 1:** State-of-the-art Ge-on-Si and InGaAs/InP SPADs.

Year	Platform	Temperature (K)	DCR (kHz)	Wavelength (nm)	SPDE (%)	NEP (WHz^−1/2^)	Integrated
2017 [25]	Ge-on-Si	80	534	1310	27.09^a^	6.2 × 10^−16^	Yes
2020 [43]	Ge-on-Si	125	36.9	1310	29.4%	7.7 × 10^−17^	No
2022 [44]	Ge-on-Si	300	1100	1550	21.24^a^	8.8 × 10^−16^	Yes
2022^b^	InGaAs/InP	230	2.5	1310	34	3.2 × 10^−17^	No
This work	Ge-on-Si	78	279	1310	34.62^a^	3.27 × 10^−16^	Yes

^a^On-chip detection efficiency after subtracting the loss of grating coupler. ^b^Data from micro photon devices Srl. PDM-IR 25 µm diameter InGaAs/InP SPAD.

**Figure 8:**
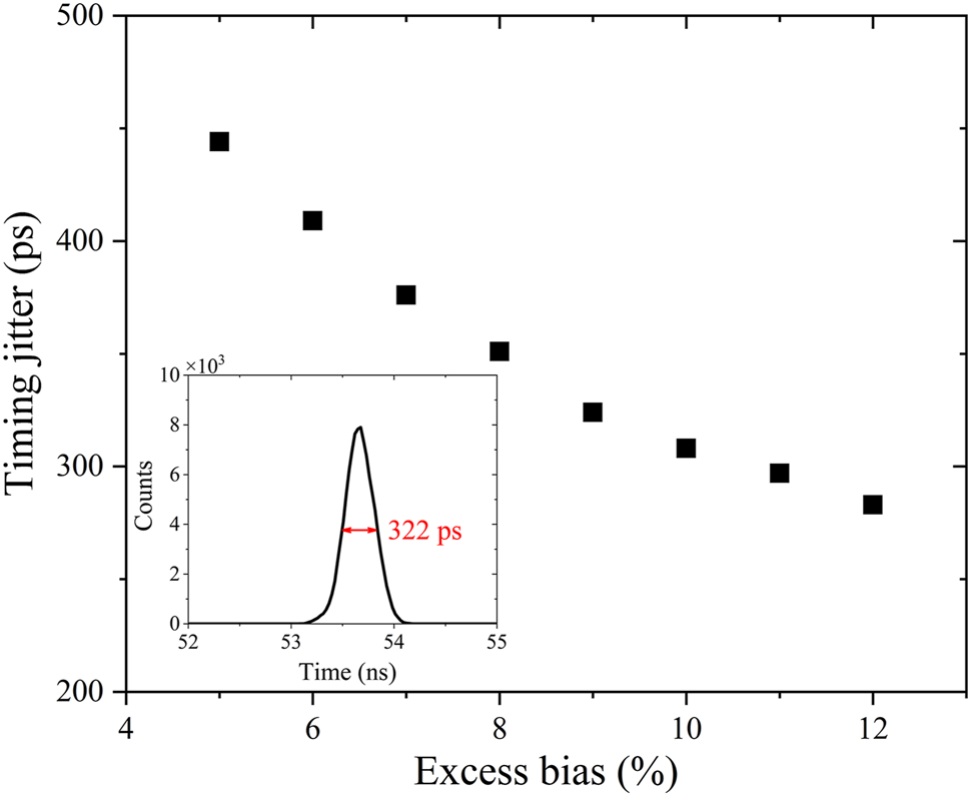
The measured system timing jitter as a function of excess bias at *V*_S1_ = 1 V. The inset shows the time histogram for the lowest jitter obtained at 12% excess bias.

Finally, the timing jitter is measured from the TCSPC histograms. Through Gaussian fitting of the time count histogram data, the timing jitter is defined as the full-width half-maximum (FWHM) of the histogram. Figure 8 shows the measured system timing jitter as a function of excess bias, which is inversely proportional to the excess bias. The inset is the measured time count histogram at 12% excess bias, the measured FWHM is about 322 ps. The larger time jitter compared with all-Si SPADs is mainly due to the inability of carriers to drift at saturation speed in the Ge absorption layer caused by the weak electric field [27].

Even though the Ge-on-Si SACM SPAD exhibits such good performances, there are still some challenges remain to be overcome. Due to the 800 nm wide intrinsic Si, the operating voltage for the SPAD is quite high, which makes it challenging for CMOS circuitry. This issue can be resolved by reducing the width of intrinsic Si appropriately. In this case, a trade-off between the intrinsic silicon width and detection efficiency of the device needs to be well considered since the avalanche probability is also decreased. On the other hand, the DCR is still higher than that of the InGaAs/InP SPADs. Also, reaching the saturated avalanche probability before the device breaks down is still challenging in the experiment, which hinders the further improvement of the detection efficiency. Reducing the noise level and increasing the avalanche probability through optimizing the device structure may lead to an improvement on the overall performance of SPADs. One method is to reduce the device active area of Ge and the reduced absorption can be enhanced by optical structures such as photonic crystals or micro rings. Other design such as suppressing dark current by doping in the intrinsic Si is also a demonstrated way [44]. Another limitation is the relatively low working temperature. In order to suppress DCRs, Ge-on-Si SPADs are usually operated at 78 K–200 K. However, the cut-off wavelength of the Ge absorber experiences a blue shift as the temperature decreases. Thus, at the optical communication bandwidth around 1550 nm, photons absorbed by Ge are significantly reduced under the low temperature condition, leading to a degraded detection efficiency of Ge-on-Si SPADs. A good way currently being explored is to use materials with high absorption in the near-infrared band to replace Ge absorber, such as Germanium-tin (GeSn). However, the fabrication is still challenging due to the poor thermal stability and the large lattice mismatch with Si [45–47].

## 4 Conclusions

We report a high-performance waveguide coupled Ge-on-Si SACM SPAD with independently controllable absorption and multiplication. By providing three electric terminals and two separate voltage drops on the absorption and multiplication regions, the electrical field in the Ge absorption layer can be adjusted separately. With controlling the voltage and reducing the electric field in Ge absorption region, the DCR can be well reduced without affecting SPDE. In addition, the three-terminal SPAD exhibits lower design difficulty and complexity compared with the conventional SACM SPADs. The presented high-performance SPAD achieves a 3.27 × 10^−16^ WHz^−1/2^ NEP with excess bias of 12%, an on-chip SPDE of 34.62%, and a DCR of 279 kHz. It shows the potential for on-chip applications such as lidar and quantum technology applications.
